# Knockdown of ANXA10 induces ferroptosis by inhibiting autophagy-mediated TFRC degradation in colorectal cancer

**DOI:** 10.1038/s41419-023-06114-2

**Published:** 2023-09-04

**Authors:** Xinyuan Wang, Yujie Zhou, Lijun Ning, Jinnan Chen, Huimin Chen, Xiaobo Li

**Affiliations:** Division of Gastroenterology and Hepatology, Shanghai Institute of Digestive Disease, NHC Key Laboratory of Digestive Diseases, State Key Laboratory for Oncogenes and Related Genes, Renji Hospital, School of Medicine, Shanghai Jiao Tong University, Shanghai, China

**Keywords:** Colon cancer, Diagnostic markers, Tumour biomarkers

## Abstract

Annexin A10 (ANXA10) belongs to a family of membrane-bound calcium-dependent phospholipid-binding proteins, but its precise function remains unclear. Further research is required to understand its role in sessile serrated lesions (SSL) and colorectal cancer (CRC). We conducted transcriptome sequencing on pairs of SSL and corresponding normal control (NC) samples. Bioinformatic methods were utilized to assess ANXA10 expression in CRC. We knocked down and overexpressed ANXA10 in CRC cells to examine its effects on cell malignant ability. The effect of ANXA10 on lung metastasis of xenograft tumor cells in nude mice was also assessed. Furthermore, we used quantitative polymerase chain reaction, western blotting, and flow cytometry for reactive oxygen species (ROS), lipid ROS, and intracellular Fe^2+^ to measure ferroptosis. Immunoblotting and Immunofluorescence staining were used to detect autophagy. We found that ANXA10 was significantly overexpressed in SSL compared to NC. ANXA10 was also highly expressed in *BRAF* mutant CRCs and was associated with poor prognosis. ANXA10 knockdown reduced the survival, proliferation, and migration ability of CRC cells. Knockdown of ANXA10 inhibited lung metastasis of CRC cells in mice. ANXA10 knockdown increased transferrin receptor (TFRC) protein levels and led to downregulation of GSH/GSSG, increased Fe^2+^, MDA concentration, and ROS and lipid ROS in cells. Knockdown of ANXA10 inhibited TFRC degradation and was accompanied by an accumulation of autophagic flux and an increase in SQSTM1. Finally, Fer-1 rescued the migration and viability of ANXA10 knockdown cell lines. In brief, the knockdown of ANXA10 induces cellular ferroptosis by inhibiting autophagy-mediated TFRC degradation, thereby inhibiting CRC progression. This study reveals the mechanism of ANXA10 in ferroptosis, suggesting that it may serve as a potential therapeutic target for CRC of the serrated pathway.

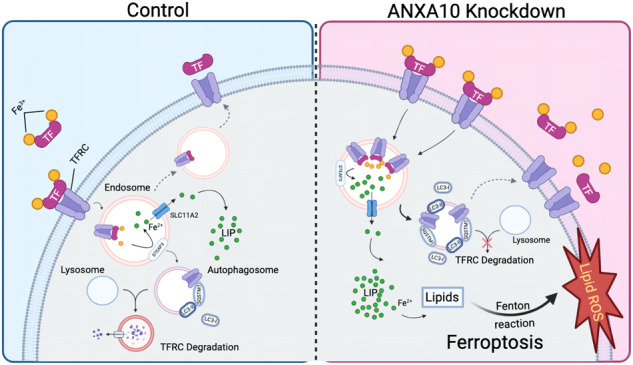

## Introduction

Colorectal cancer (CRC) is the third most commonly diagnosed cancer worldwide and the second leading cause of cancer death for both sexes [1]. In addition to the traditional adenoma-carcinoma sequence, the serrated pathway is the other major pathway leading to CRC. This pathway is characterized by the presence of a serrated lesion (SL) as a precancerous lesion [2]. However, previously, colorectal SL was misinterpreted as a harmless lesion and classified as hyperplastic polyps (HPs) [3]. In the last decade, intensive research has revealed that SL is one of the leading causes of “interstitial cancer”. Approximately 25% of sporadic CRCs develop through SL [4]. According to the latest WHO classification of gastrointestinal tumors (5th edition) published in 2019, colorectal SL is classified into four categories: HPs, sessile serrated lesions (SSL), and sessile serrated lesions with dysplasia (SSLD), traditional serrated adenomas (TSAs) and unclassified SL [5].

Of these, SSL is thought to have malignant potential, progressing rapidly to CRC in a short period. Two case reports have reported rapid progression to invasive tumors at 8 and 13 months, respectively [6, 7]. SSL is easily overlooked during colonoscopy as they present as pale, ill-defined flat lesions with a mucous surface often adhering in a “cloudy” appearance. This feature allows SSL to escape detection and further contributes to their progression [8]. At the molecular level, the *BRAF*^*V600E*^ mutation and the CpG island methylator phenotype-high (CIMP-H) are the two main features of SSL and the sessile serrated carcinoma pathway [9, 10]. However, the mechanisms of SSL and serrated carcinoma pathway development remain poorly understood.

Annexin A10 (ANXA10, A10) is a member of the calcium-dependent phospholipid-binding protein family, whose function has not yet been determined [11]. Several studies have found that ANXA10 is specifically highly expressed in colon SSL and MSI-H CRC [12–16], but its mechanism of action has not been investigated in depth. In other different cancers, ANXA10 has been associated with distinct roles, with high expression in some cancers, such as melanoma [17], extrahepatic cholangiocarcinoma [18], esophageal epithelial squamous cell carcinoma [19], and oral cancer [20] being associated with worse prognosis, whereas it acts as a protective factor in other cancers, such as liver cancer [21], prostate cancer [22], and bladder cancer [23]. Therefore, the role of ANXA10 in SSL and CRC needs to be further investigated.

In this study, ANXA10 was identified as a molecule differentially expressed in SSL and the serrated pathway by transcriptome sequencing. The specific molecular mechanisms of ANXA10 were investigated to seek out potential targets for intervention, paving the way for precise prevention and treatment of CRC.

## Methods and materials

### Patient samples

This study utilized 12 pairs of sessile serrated colorectal lesions, matched normal control specimens, and colon cancer specimens obtained from Renji Hospital, Shanghai Jiao Tong University School of Medicine. The pathological diagnosis of these lesions was conducted in accordance with the latest edition of the WHO diagnostic criteria for serrated lesions in 2019 [5]. All clinical specimens underwent postoperative pathological confirmation. Our study was approved by the Ethics Committee of Renji Hospital, Shanghai Jiao Tong University School of Medicine (KY2021-004). All patients provided informed consent.

### Antibodies and chemicals

Antibodies against ANXA10 (ab213656), β-Actin (ab8227), LC3B (ab192890), and SQSTM1 (ab109012) were purchased from Abcam (USA). Rabbit anti-β-Tubulin (#2128) antibody was purchased from Cell Signaling Technology. Rabbit anti-TFRC (10084-2-AP) was purchased from Proteintech, and mouse anti-Flag (DYKDDDDK-Tag) was purchased from Abmart. LAMP1 antibody (sc-20011) was purchased from Santa Cruz (CA, USA). Alexa Fluor 488 (A-21202) and Alexa Fluor 594 (A-21207) conjugated secondary antibodies were purchased from Invitrogen (CA, USA). Ferrostatin-1 (HY-100579), Deferoxamine mesylate (HY-B0988), Rapamycin (HY-10219), and Chloroquine (HY-17589A) were purchased from MedChemExpress. Cycloheximide (S7418) was purchased from Selleck, and DMSO (D2650) and N-acetyl-L-cysteine (A9165) were purchased from Sigma-Aldrich. Hydrogen peroxide solution (88597) was purchased from Millipore.

### Cell death, viability, and proliferation assays

Cell death was assessed using either the Annexin V-FITC Apoptosis Detection Kit I (BD Biosciences, USA) or the Annexin V-PE/7-AAD Apoptosis Detection Kit (Vazyme, Nanjing, China) in accordance with the manufacturer’s instructions. The proportion of cell death was subsequently measured by flow cytometry. Cell viability was determined using the Cell Counting Kit-8 (CCK-8) (Dojindo, Japan) as per the manufacturer’s recommendation. To measure cell proliferation, the CCK-8 (Dojindo, Japan) was used following the manufacturer’s instructions.

### Cell culture, transfection, and generating stable cell lines

The human colorectal cancer cell lines used in this study (HT29, HCT116, SW480, and RKO) were obtained from American Type Culture Collection (ATCC). Of these, HT29 and RKO cells carry the *BRAF* V600E mutation, while HCT116 and SW480 cells have the wild-type *BRAF* gene. HT29 and HCT116 cells were cultured in McCoy'5a medium, while SW480 and RKO cells were cultured in RPMI 1640 medium (both from Gibco, USA). In addition, 10% fetal bovine serum and 1% penicillin–streptomycin (both from Gibco, USA) were added to the respective media. All cell lines were incubated at 37 °C in a 5% CO_2_ atmosphere.

The day before transfection, well-grown cells were seeded onto plates following the manufacturer’s instructions. Once the cells reached 60–80% confluence, small-interfering RNAs (siRNAs) and the plasmid were transfected using DharmaFECT 1 siRNA transfection reagent (Horizon Discovery Ltd., USA) and FuGene HD transfection reagent (Promega, Madison, WI, USA), respectively. The siRNAs were purchased from GenePharma Technology (Shanghai, China), while the plasmid and LV-ANXA10-shRNA were obtained from GeneChem (Shanghai, China). To achieve knockdown, Lentiviral-packed shRNA was used to infect HT29 cells with HiTransG P according to the manufacturer’s protocol. After 72 h, positive cells were selected using puromycin (8 μg/ml) for 2–3 generations to establish cell lines that were stably infected with shRNA. The plasmid and shRNA constructs used were GV492 and GV493 and the sequences of siRNAs and shRNA are listed in Table S1.

### In vivo mouse xenograft models

For the in vivo xenograft mouse model, 5-week-old BALB/c male nude mice were purchased from GemPharmatech Co. Ltd. (Nanjing, China) and kept in specific pathogen-free conditions at 25 °C with suitable humidity and a light/dark cycle. To create a single-cell suspension, 1 × 10^6^ shNC or shANXA10 HT29 cells were dissolved in 200 μL PBS and mixed. The mice were randomly divided into two groups (*n* = 5 per group) and injected with the single-cell suspension through the tail vein. After eight weeks, the mice were sacrificed, and their lung tissues were extracted, observed, and photographed. The tissues were fixed in formalin for 24 h, embedded in paraffin, and stained with H&E. All study procedures were approved by the Institutional Animal Care and Use Committee of Renji Hospital, School of Medicine, Shanghai Jiaotong University.

### Immunohistochemistry (IHC)

SSL, CRC, and normal colon tissues were obtained from Renji Hospital, Shanghai Jiao Tong University School of Medicine, and all samples were formalin-fixed and embedded in paraffin tissue blocks. The tissue blocks were sectioned into 4 μm slices. After deparaffinization and antigen retrieval, the sections were incubated with 3% hydrogen peroxide solution for 20 min to quench endogenous peroxidase activity and with goat serum for 30 min to block nonspecific binding. Next, the sections were incubated overnight at 4 °C with an anti-human ANXA10 antibody (1:200) and for 30 min at room temperature with a horseradish peroxidase-conjugated secondary antibody. The sections were then visualized with 3,3-diaminobenzidine substrate and counterstained with hematoxylin before observation under a light microscope.

### Immunofluorescence staining

HT29 cells were seeded at a density of 2 × 10^4^ cells/well into a Nunc™ Lab-Tek™ II 8-well Chambered Slide (Thermo Fisher Scientific, Rochester, NY, USA) and transfected with siRNA after 24 h. Cells were further cultured for 48 h and then fixed with 4% paraformaldehyde for 30 min. Cells were washed three times in PBS, permeabilized by 0.3% Triton X-100 for 30 min, and blocked using 1% BSA in PBS for 30 min. Cells were then incubated with primary antibodies against TFRC (1:100) and LAMP1 (1:100) overnight at 4 °C. The slide was incubated with Alexa Fluor 488- and 594-conjugated secondary antibodies for 1 h at room temperature, and followed by staining with DAPI (Southern Biotech, Birmingham, AL). Fluorescence images were photographed with FV3000 laser confocal microscope (Olympus, Japan).

### Transcriptome sequencing (RNA-seq) analysis

Total RNAs were extracted from SSL, paired normal tissues, HT29-shANXA10, and HT29-shNC cells using Trizol (Invitrogen, Carlsbad, CA, USA) according to the protocol. The RNAs were subsequently qualified and quantified using NanoDrop 2000 (Thermo Fisher Scientific, MA, USA). Purified mRNA was randomly fragmented into small fragments using Oligo(dT)-attached magnetic beads at the appropriate temperature and subjected to PCR reactions to synthesize first-strand cDNA and second-strand cDNA. The reaction products were then purified using magnetic beads, followed by the addition of A-Tailing Mix and RNA Index Adapters to repair the ends and subsequent PCR amplification. The final libraries were obtained by purifying the products using Ampure XP magnetic beads, which were then assessed for quality and quantity using an Agilent 2100 bioanalyzer before undergoing DSN treatment. The DSN-treated libraries were evaluated for quality in two ways: (i) by checking the distribution of fragment sizes using the Agilent 2100 bioanalyzer and (ii) by quantifying the libraries using qPCR. Single-end sequencing was performed on the Illumina Novaseq 6000 platform (Tsingke, Beijing, China).

To examine the coherence of samples within groups and the differences in variability between groups in RNA-seq, we employed principal component analysis (PCA) to analyze all samples. The analysis and visualization of results were carried out using the “FactoMineR” and “factorextra” R packages. For the identification of differentially expressed genes (DEG), we conducted an enrichment analysis of the Kyoto Encyclopedia of Genes and Genomes (KEGG) signaling pathway using the “clusterProfiler” R package. The enrichment results were then presented graphically using the “enrichplot” R package.

### Single-cell transcriptome sequencing analysis

The different types of intestinal epithelial cells were clustered and grouped using the R package “Seurat” (version 4.0.3). Other analysis steps were based on our previous study [24]. Ultimately, we discovered eight significant epithelial cell types in the SL, including a specific subpopulation unique to the SL, which we named Epi-SL. We then compared the expression levels of ANXA10 in the eight distinct cell subpopulations.

### Quantitative real-time polymerase chain reaction (qPCR)

To extract total RNA from cells, we utilized Trizol (Invitrogen, Carlsbad, CA, USA) according to the manufacturer’s instructions. The extracted RNA was assessed for quality and quantity using NanoDrop 2000 (Thermo Fisher Scientific, MA, USA), followed by reverse transcription to cDNA using a PrimeScript RT Reagent Kit (Takara, Japan). Quantitative RT-PCR was then conducted using SYBR Premix Ex Taq (Takara, Japan) in accordance with the manufacturer’s guidelines. The expression levels of mRNAs were normalized to β-Actin. Table S2 contains the sequences of all primers used in the experiment.

### Wound-healing assay and transwell assay

Wound-healing assays and transwell assays were performed to assess the migration capacity of the cells. For the wound-healing assay, cells were plated in 6-well plates at a density of 4 × 10^5^ cells per well and transfected with plasmids the following day. Once the cells reached 90% confluence, they were vertically scratched in the plate using a sterile 200 μL gun tip. After washing twice with PBS, the cells were cultured in a serum-free medium. Microscopic images were captured at 0 h and 40 h, and the area of the scratched region was calculated relative to the 0 h time point to determine the migration rate.

For the transwell assay, 600 μL of 30% FBS medium was added to a 24-well plate, and transwell chambers (Millipore, Darmstadt, Germany) were placed on top. Next, 300 μL of cell suspension at a concentration of 1 × 10^6^ cells/mL was added to each chamber. The cells were then incubated for 24–72 h, depending on the characteristics of the cells. After incubation, the 24-well plate was removed, and the upper chamber medium was discarded. The cells on the upper surface of the membrane were wiped off using a cotton swab, and the outer layer of the chambers was washed twice with PBS. The cells were then fixed using 600 μL of 4% paraformaldehyde for 30 min, followed by staining with 600 μL of crystalline violet staining solution for 15 min at room temperature. After washing with PBS, the cells were air-dried and observed under a microscope. Six randomly selected fields of view were photographed, and the number of cells was counted.

### Measurement of lipid peroxidation and reactive oxygen species (ROS)

Cells were inoculated in plates and transfected with siRNA after 24 h. To detect levels of lipid peroxidation, the old medium was removed after 48 h of incubation, and the cells were washed once with a serum-free medium. Then, 500 μL of a 10 μM Liperfluo solution (#L248, Dojindo, Japan) diluted with serum-free medium was added to each well and incubated for 30 min at 37 °C. The cells were washed twice with serum-free medium, and then digested with trypsin. After centrifuging and resuspending with 300 μL of PBS, the fluorescence intensity of the samples was measured using flow cytometry.

To detect ROS levels, the old medium was removed after continuing incubation for 48 h, and 500 μL of a 10 μM DCFH-DA (#S0033, Beyotime Biotechnology, China) working solution prepared with serum-free medium was added to each well and incubated for 20 min at 37 °C. The cells were then washed three times with serum-free medium, and digested with trypsin, and the fluorescence intensity of the samples was determined using flow cytometry.

### Immunoblotting

Whole cells were harvested and lysed in RIPA buffer supplemented with protease and phosphatase inhibitors. The lysates were then separated by SDS-PAGE and transferred onto nitrocellulose membranes. The membranes were blocked with a blocking solution, and then incubated overnight at 4 °C with primary antibodies. After washing, the membranes were incubated for 1 h at room temperature with secondary antibodies. The proteins of interest were detected by chemiluminescence using ChemiDoc Touch (Bio-Rad, California, USA). All the original western blot images were shown in supplementary information.

### Glutathione (GSH) quantification and malondialdehyde (MDA) assay

Intracellular levels of all glutathione and oxidized glutathione (GSSG) were detected using the GSH/GSSG Detection Kit (#S0053, Beyotime Biotechnology, China) according to the manufacturer’s instructions. The intracellular MDA concentration was determined using a Cell Malondialdehyde Assay Kit (Colorimetric method, #A003-4-1, Nanjing Jiancheng Bioengineering Institute, China) according to the manufacturer’s recommended protocol.

### Cell ferrous ion (Fe^2+^) level analysis

After removing the supernatant from the plate, the cells were washed three times with a serum-free medium. Subsequently, FerroOrange (1 μM, #F374, Dojindo, Japan) working solution (diluted with serum-free medium) was added and the cells were incubated for 30 min at 37 °C, as per the manufacturer’s instruction. The cells were then observed and photographed directly under a fluorescence microscope. Alternatively, cells were trypsinized, centrifuged at 1000 rpm for 5 min at 4 °C, and resuspended in 400 μL of serum-free medium. The fluorescence intensity was immediately measured using a flow cytometer.

### Mass spectrometry (MS)

After protein extraction and reductive alkylation, the samples were analyzed by MS on a Thermo Fisher LTQ Qbitrap ETD Mass spectrometer. In brief, samples were placed on a Thermo Fisher Easy-nLC 1000 HPLC system equipped with a C18 column (1.8 mm, 0.15 × 1.00 mm) with solution A containing 0.1% formic acid and solution B with 100% acetonitrile. MS was performed in positive ion mode at a flow rate of 300 nL/min. FTMS was used to obtain a full scan (350–1600 *m*/*z*) at a mass resolution of 30,000, followed by MS/MS using higher-energy collisional dissociation for the 10 most intense pioneer ions at 35% collisional energy and a mass resolution of 15,000. The raw MS files were analyzed using MaxQuant (1.6.1.0). The search included dynamic modifications of methionine oxidation and deamidation and static modification of carbamidomethyl cysteine. A maximum of two missed cleavages was allowed, and the mass tolerance for precursor ions and fragment ions was set to 20 ppm and 4.5 ppm, respectively. The minimal peptide length was set to six amino acids, and a maximum of two miscleavages was allowed. The peptide and protein identifications were accepted at a false discovery rate of 0.01.

### Protein degradation assay

Cells were pre-inoculated in 6-well plates with a density of 5 × 10^5^ cells per well. The old medium was then removed, and 2 mL of the complete medium was added to each well. Cycloheximide (CHX) was added to each well at pre-set time points of 12th, 9th, 6th, and 0th hour according to the pre-set incubation time gradient of CHX (0, 3, 6, and 12 h). Therefore, we added 2 μL of 50 mg/mL of CHX to the corresponding wells at each pre-set time point, respectively, resulting in a final concentration of 50 μg/mL. After a total incubation period of 12 h, all samples were subjected to protein extraction, followed by immunoblotting analysis to determine the relative gray values of the target proteins, with β-Actin expression serving as the internal reference.

### Public database analysis

The mRNA transcriptome sequencing data for Colon Adenocarcinoma (COAD) from The Cancer Genome Atlas (TCGA, http://cancergenome.nih.gov) and clinical information were obtained from the UCSC Xena database (https://xena.ucsc.edu/). The transcriptome data were converted into log2CPM (counts per million) for subsequent analysis using the “edgeR” R package. The colon cancer mRNA microarray dataset GSE39582, which includes 51 *BRAF* mutant CRC patients and 474 *BRAF* wild-type CRC patients, from the Gene Expression Omnibus (GEO) database was obtained using the “GEOquery” R package and annotated using the “clusterProfiler” R package. The survival analysis of CRC patients was done using the “survival” and “survminer” R software packages, and the cutoff value for the grouping of ANXA10 expression levels was determined using the X-tile software. The relationship between gene expression levels and clinical information was processed using the “reshape2” R package and visualized using the “ggpubr” R package.

### Gene set variation analysis

GSVA analysis is used to assess the enrichment of a gene set in individual samples. This analysis was performed using the “GSVA” R package, which calculates the enrichment scores, and the “ggplot2” R package was used to visualize the data.

### Statistical analysis

All statistical analyses were performed using GraphPad Prism 9.3.1 or R 4.1.2 software. For statistical analysis between two groups of samples, an independent sample *t-*test was used if they obeyed a normal distribution, while paired samples used a paired *t-*test. For three or more groups of samples, the one-way ANOVA was performed. Non-parametric tests were used for data that did not follow a normal distribution. All experiments were repeated three times, and differences were considered significant if the *P-*value < 0.05.

Flow cytometry data were analyzed using FlowJo 10.4.0 software. All images were assessed using ImageJ 1.53 software.

## Results

### ANXA10 is upregulated in the serrated pathway

**Fig. 1:**
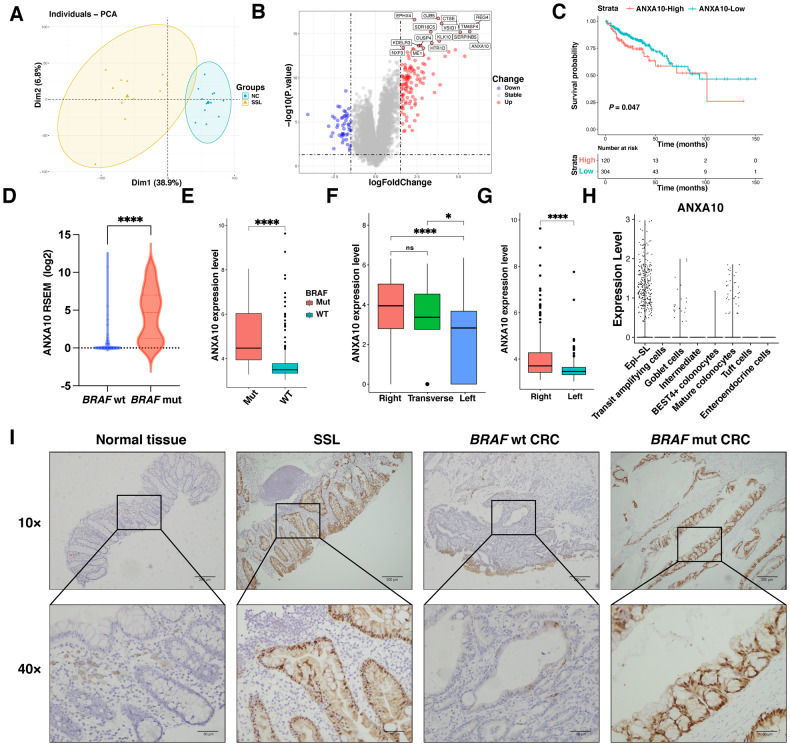
ANXA10 is specifically overexpressed in the serrated pathway and is associated with poor prognosis. **A** PCA analysis of 12 pairs of SSL and their corresponding NC transcriptomes sequenced showed significant differences in transcriptome levels between them. **B** Volcano plot of DEG between SSL and NC. ANXA10 was significantly overexpressed in SSL (red arrow). The names of the 15 genes with the lowest *P*-values are marked in the graph. **C** Kaplan–Meier analysis of OS revealed that the higher the level of ANXA10 expression, the worse the prognosis of CRC patients in the TCGA-COAD database. **D**, **E** ANXA10 was highly expressed in *BRAF*mut CRC patients compared to in *BRAF*wt. TCGA-COAD (**D**); GSE39582 (**E**). **F**, **G** ANXA10 was expressed at distinctly higher levels in CRC located in the right hemicolectomy than in the left. TCGA-COAD (**F**); GSE39582 (**G**). **H** In the serrated lesion single-cell sequencing data, ANXA10 was highly expressed in Epi-SL. **I** Representative images of immunohistochemical staining, with ANXA10 specifically overexpressed in the epithelial crypt at the SSL and BRAFmut CRC lesions site. The scale bars are 200 μm (10×) and 50 μm (40×). *P < 0.05,**P < 0.01,***P < 0.001,****P < 0.0001. PCA principal component analysis, SSL sessile serrated lesion, NC normal control, OS overall survival, DEG differentially expressed genes, Epi-SL serrated lesion-specific epithelial cell.

To investigate the features of the serrated pathway, we conducted transcriptome sequencing (RNA-seq) on twelve pairs of SSL and corresponding NC. PCA analysis revealed significant differences between the SSL and NC groups (Fig. 1A). We performed differential analysis and identified differently expressed genes (DEG) using the |log2FoldChange|value > 1.5 and *P* < 0.05 criteria. ANXA10 was found to be significantly upregulated in SSL (Fig. 1B).

Given the high expression of ANXA10 in SSL, we further examined its expression levels in CRC patients. Analysis of the TCGA-COAD and GEO databases revealed that CRC patients with high ANXA10 expression had worse prognoses than those with low expression (*P* = 0.047) (Fig. 1C). In addition, ANXA10 expression was substantially higher in *BRAF*-mutated CRC (*BRAF*mut CRC) than in *BRAF* wild-type (*BRAF*wt CRC) (*P* < 0.0001; Fig. 1D, E). Furthermore, ANXA10 was expressed at higher levels in CRC located in the right colon than in the left (Fig. 1F, G), consistent with the fact that SSL and *BRAF*-mutated CRC occur more frequently in the right.

We also performed single-cell transcriptome sequencing of serrated lesions and discovered a subpopulation of epithelial cells specific to serrated lesions (Epi-SL) (Fig. 1H) [24]. Interestingly, ANXA10 was highly expressed in Epi-SL, while its expression was lower in normal subpopulations such as absorptive epithelium and cupped cells. Immunohistochemical staining revealed that ANXA10 was specifically upregulated in the crypt at the site of lesions in SSL and *BRAF*mut CRC (Fig. 1I).

### Knockdown of ANXA10 represses viability and promotes the death of colorectal cancer cells

**Fig. 2:**
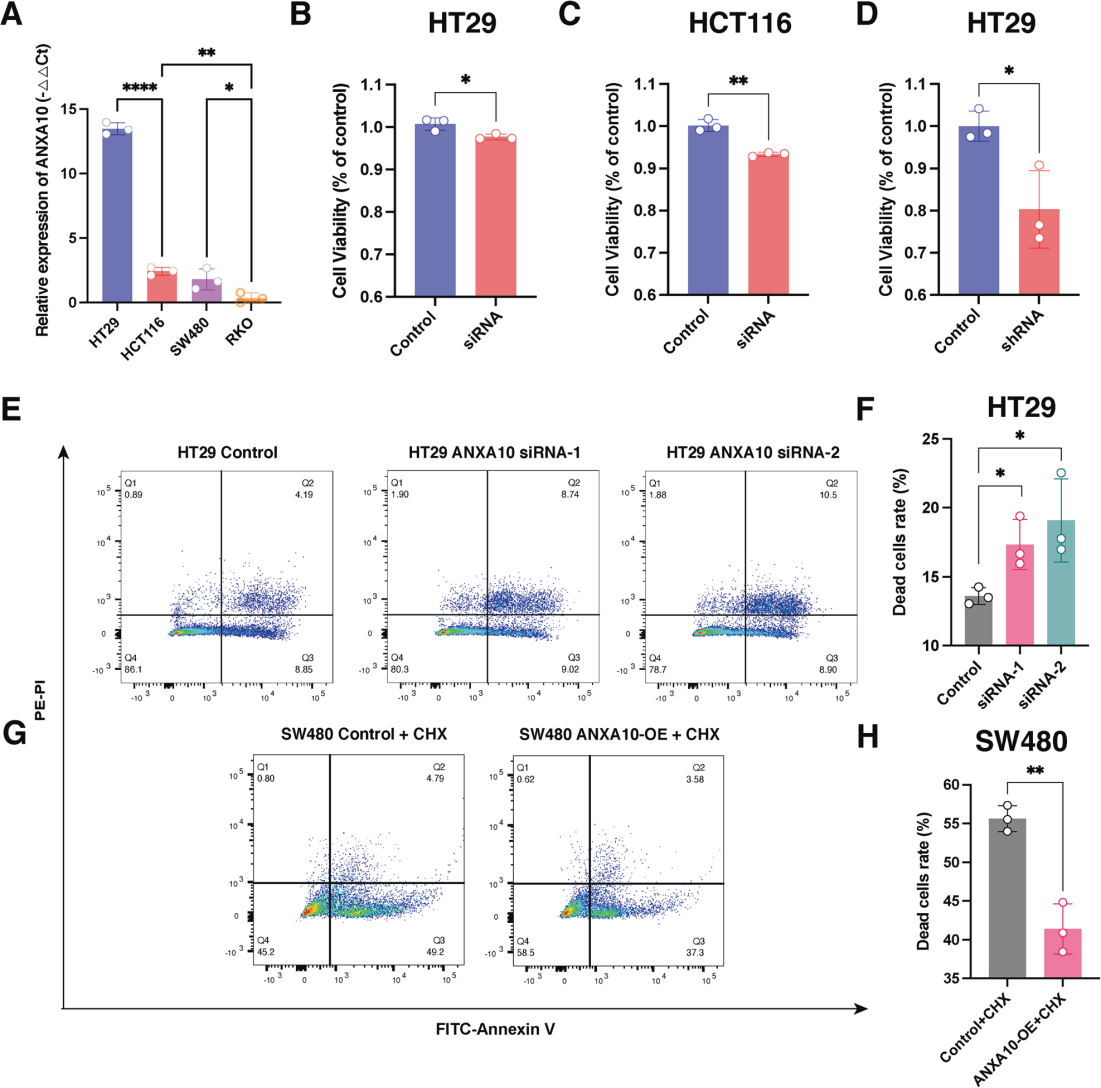
Knockdown ANXA10 reduced colorectal cancer cell survival and promoted apoptosis. **A** Expression levels of ANXA10 mRNA in different CRC cell lines. **B**–**D** CCK-8 assay to detect the survival rate of HT29 (**B**) and HCT116 (**C**) cells after transfection with siRNA and HT29 cells (**D**) after infection with lentiviral-packed shRNA. **E**, **F** Flow plots of the change in the percentage of apoptosis (**E**) and statistical results (**F**) of the percentage of early apoptosis + late apoptosis (Q2 + Q3) after knockdown of HT29 cells using flow cytometry. **G**, **H** Flow plots of the change in the percentage of apoptosis (**G**) and statistical results (**H**) of the percentage of early apoptosis + late apoptosis (Q2 + Q3) after overexpression of ANXA10 and treatment with 5 μg/ml CHX for 24 h in SW480 cells using flow cytometry. *P < 0.05, **P < 0.01. CCK-8 Cell Counting Kit-8; CHX cycloheximide.

To investigate the impact of ANXA10 on CRC development, we analyzed the expression levels of ANXA10 mRNA in various CRC cell lines and found the highest expression in HT29 cells, followed by HCT116 and SW480 cells, and the lowest expression in RKO cells (Fig. 2A). To study the role of ANXA10 in CRC, we performed knockdown experiments of ANXA10 in HT29 and HCT116 cells and overexpression experiments in SW480 cells.

We used small-interfering RNAs (siRNAs) to knock down ANXA10 expression in HT29 and HCT116 cells, and siRNA-2 was identified as the most effective siRNA for ANXA10 knockdown, as confirmed by RT-PCR and Western blot analysis (Fig. S1A, B). Meanwhile, we transfected ANXA10-shRNA and control lentiviral plasmids into HT29 cells to construct ANXA10 knockdown (shANAX10) and control HT29 stable-transformed cell lines (shNC), respectively, and confirmed the knockdown efficiency by Western blot and RT-PCR analysis (Fig. S1C). Furthermore, we transiently transfected ANXA10 overexpression plasmids in SW480 and RKO cells and verified ANXA10 overexpression (Fig. S1D, E).

We then examined the effects of ANXA10 on cell survival and apoptosis. The viability of HT29 and HCT116 was significantly inhibited by ANXA10 siRNA (Fig. 2B, C), and a similar decrease in cell viability was observed in the HT29-shANXA10 stable-transformed cell line (Fig. 2D). Moreover, the knockdown of ANXA10 led to a significant increase in the proportion of early and late apoptotic cells (Fig. 2E, F and S2A, B), whereas ANXA10 overexpression in SW480 and RKO cells significantly decreased the percentage of apoptotic cells with or without cycloheximide (CHX) (Fig. 2G, H and S2C–H).

### ANXA10 enhances the proliferation and migration of CRC cells

**Fig. 3:**
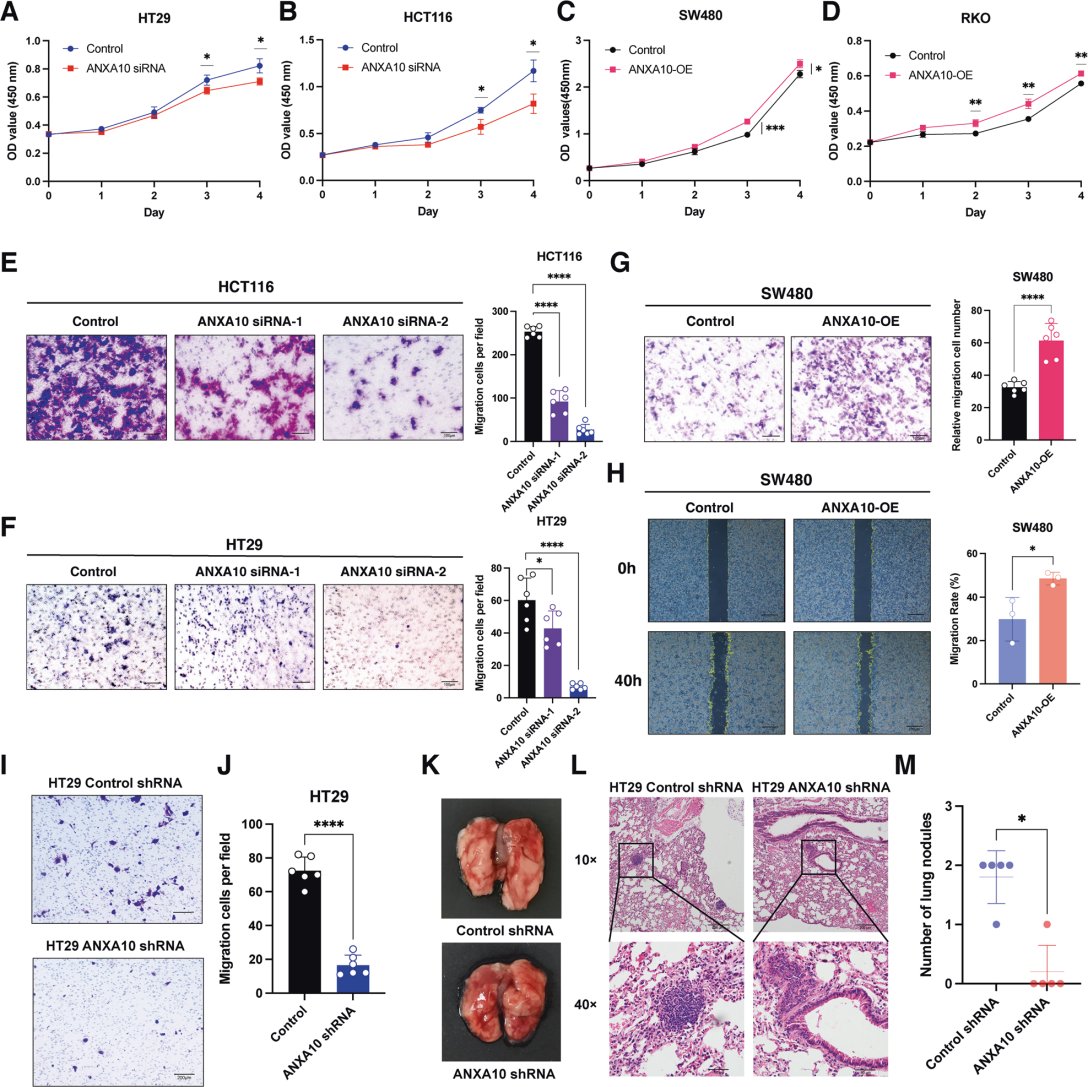
ANXA10 promoted the proliferation and migration of colorectal cancer cells. **A**, **B** CCK-8 assay to determine the level of proliferation and found decreased proliferation capacity after knockdown of ANXA10 in HT29 (**A**) and HCT116 (**B**) cells. **C**, **D** CCK-8 assay to determine the level of proliferation and found increased proliferation capacity after overexpression of ANXA10 in SW480 (**C**) and RKO (**D**) cells. **E**, **F** Transwell migration assay to detect the effect of knockdown of ANXA10 on the migration ability of HCT116 (**E**) and HT29 (**F**) cells. **G** Transwell migration assay to detect the effect of overexpression of ANXA10 on the migratory ability of SW480 cells. Six randomly selected fields of view were photographed for each group; representative images are shown on the left, the scale bar is 100 μm; and statistical results are shown on the right (*n* = 6). **H** Scratch assay was performed to detect the migration ability of SW480 in control and overexpression groups. Representative images are shown on the left, scale bar is 200 μm; the migration rates of cells in each group are shown on the right. **I**, **J** Transwell migration assay was performed to detect the migration ability of the HT29-shANXA10 cell line and control group. Representative images at a scale of 200 μm (**I**) and the statistical results (*n* = 6) (**J**) were shown. **K**–**M** A lung metastasis model was constructed by injecting HT29-shANXA10 and controls into nude mice. Representative lung tissue images (**K**) and H&E-stained images of lung metastases (**L**) were illustrated at a scale of 200 μm for 10× images and 50 μm for 40× images; the statistical analysis of the number of lung metastases in the two groups is shown (**M**) (*n* = 5). *P < 0.05, **P < 0.01,***P < 0.001, ****P < 0.0001.

The CCK-8 assay showed that after the knockdown of ANXA10, HT29, and HCT116 cells proliferated significantly slower than the control group on days 3 and 4 (Fig. 3A, B). Furthermore, we observed that SW480 and RKO cells overexpressing ANXA10 proliferated substantially faster than the control cells (Fig. 3C, D).

Considering the association between high ANXA10 expression and poor prognosis in CRC patients, we speculated that ANXA10 might regulate the migration ability of CRC cells. Our results demonstrated that the knockdown of ANXA10 impaired the migratory ability of CRC cells (Fig. 3E, F), while overexpression of ANXA10 promoted their migration (Fig. 3G). Scratch assays also verified that overexpression of ANXA10 facilitated the ability of SW480 cells to heal scratches (Fig. 3H).

To further confirm the in vitro findings, we verified the migration ability of HT29-shANXA10 cells in vitro and found that it was significantly weaker than that of the control group (Fig. 3I, J). Subsequently, we established a lung metastasis model in nude mice by tail vein injection of HT29-shANXA10 or HT29-shNC cells. Eight weeks later, the mice were euthanized and found to have significantly fewer metastatic lesions in the lungs of the shANXA10-injected group compared to the control group, as confirmed by H&E staining (Fig. 3K–M).

### Ferroptosis occurred in ANXA10 knockdown colorectal cancer cells

**Fig. 4:**
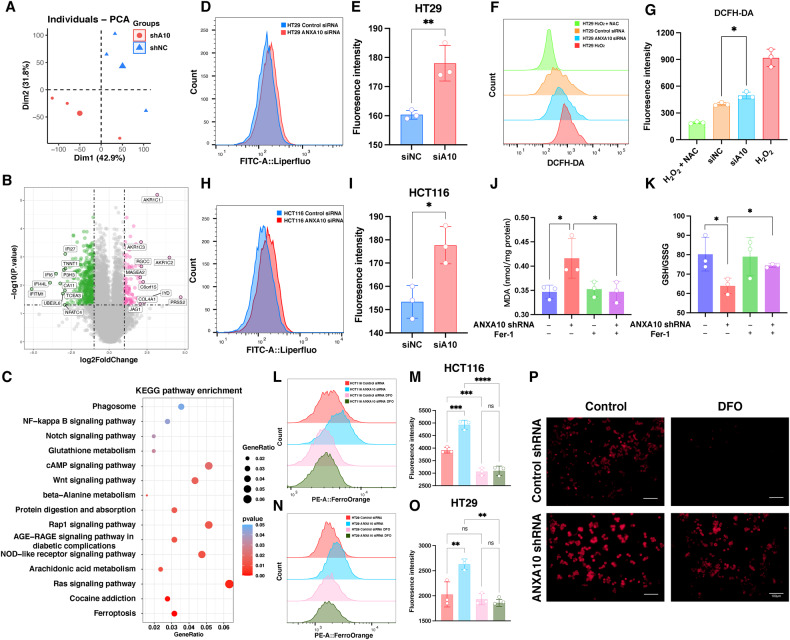
Knockdown of ANXA10 induced ferroptosis in colorectal cancer cells. **A** PCA analysis of HT29-shANXA10 and shNC transcriptome sequencing. **B** Volcano map of differentially expressed genes. The names of the 20 genes with the largest |log2FoldChange| values are marked. **C** Results of KEGG pathway enrichment analysis. **D**, **E** After the transfection of siRNA, lipid peroxidation levels were detected in HT29 using liperfluo fluorescent probes. **H**, **I** After the transfection of siRNA, lipid peroxidation levels were detected in HCT116 cells using liperfluo fluorescent probes. **F**, **G** Detection of ROS levels in HT29 cells using DCFH-DA fluorescent probe. Cells were treated with 400 μM H_2_O_2_ for 2 h as a positive control and with 400 μM H_2_O_2_ and 8 mM NAC as a negative control. **J**–**K** Intracellular MDA levels (**J**) and the ratio of reduced GSH/GSSG (**K**) were measured in HT29-shANXA10 and control cells after treating the cells with Fer-1 (1 μM) or DMSO for 48 h, respectively. **L**–**O** Intracellular Fe^2+^ levels in HCT116 (**L**, **M**) and HT29 cells (**N**, **O**) were measured using FerroOrange fluorescent probes after transfection of siRNA knockdown ANXA10 or retreatment of cells with DFO (200 μM) for 48 h, respectively. **P** Fluorescence images of the intracellular FerroOrange probe in the HT29-shANXA10 and shNC groups at a scale of 100 μM. *P < 0.05,**P < 0.01,***P < 0.001,****P < 0.0001. PCA principal component analysis, ROS oxygen species, MDA malondialdehyde, GSSG oxidized glutathione, GSH glutathione, DFO deferoxamine mesylate, Fer-1 ferrostatin-1, NAC N-acetyl-l-cysteine, H_2_O_2_ hydrogen peroxide.

To elucidate the molecular mechanisms by which ANXA10 promotes malignant biological behaviors, such as survival, proliferation, and migration of CRC cells, we performed RNA-seq on HT29-shANXA10 and shNC cells. PCA reduced dimensional analysis revealed intergroup differences between the samples of the two groups, suggesting differences in transcriptome levels (Fig. 4A). Upon screening for DEG based on the criteria of differential expression fold (Fold-Change value) >2-fold or <0.5-fold and *P* < 0.05, we identified 153 genes with upregulated expression and 485 genes with down-regulated expression in the HT29-shANXA10 group compared to the control (Fig. 4B). KEGG pathway enrichment analysis on all up- and down-regulated genes revealed the ferroptosis pathway as the most significant among the top 15 pathways in terms of significance (*P* < 0.0001), along with other signaling pathways such as the Ras signaling pathway, arachidonic acid metabolism, NOD-like receptor signaling pathway, and Rap1 signaling pathway (Fig. 4C).

The considerable enrichment of the ferroptosis pathway prompted us to initiate a study on the role of ANXA10 in regulating this process. Lipid peroxidation and iron accumulation are two major biochemical features involved in ferroptosis. We, therefore, investigated the effect of knocking down ANXA10 on lipid peroxidation levels in CRC cells. Our results showed that after siRNA knockdown of ANXA10 in HT29 and HCT116 cells (siANXA10), the mean fluorescence intensity was significantly stronger detected by the liperfluo fluorescent probe compared to the control (*P* < 0.01 and *P* < 0.05, respectively; Fig. 4D, E, H, I). In addition, we also observed an increase in intracellular reactive oxygen species (ROS) levels in HT29 cells after ANXA10 knockdown using a DCFH-DA fluorescent probe (*P* < 0.01, Fig. 4F, G). These experimental results suggest that the lipid peroxidation and ROS levels were markedly increased after knocking down ANXA10 in CRC cells.

The primary products of lipid peroxidation include lipid peroxide (LOOH), malondialdehyde (MDA), and 4-hydroxynonenal (4HNE), and intracellular MDA concentration can indirectly reflect the degree of lipid peroxidation [25]. Our experimental results showed that the MDA concentration in the HT29-shANXA10 group was soaring ahead of the control (*P* < 0.01). Notably, treating cells with the ferroptosis inhibitor Ferrostatin-1 (Fer-1) for 48 h significantly reduced the MDA concentration in the shANXA10 group (*P* < 0.05), and there was no significant difference between the shANXA10 and control groups (Fig. 4J).

Glutathione (GSH) is the main intracellular antioxidant, and depletion of GSH exacerbates the accumulation of intracellular ROS, ultimately triggering ferroptosis [26]. Therefore, we further examined the ratio of reduced GSH to oxidized glutathione (GSSG) in cells. The results showed that the GSH/GSSG ratio was dramatically lower in the shANXA10 cohort than in the control group, while treatment with Fer-1 significantly restored the GSH/GSSG ratio in HT29-shANXA10 cells (Fig. 4K).

Subsequently, we detected the levels of Fe^2+^ in CRC cells using the FerroOrrange fluorescent probe. We found that the mean fluorescence intensity was more robust in the siANXA10 group than in the control (*P* < 0.001). After treating both groups of cells separately with the iron chelator, deferoxamine mesylate (DFO), for 48 h, we found that DFO dramatically reduced the mean fluorescence intensity of cells in the siANXA10 group (*P* < 0.0001, Fig. 4L, M). Therefore, we again examined Fe^2+^ levels in HT29 cells by flow cytometry and found experimental results consistent with those described above. Knockdown of ANXA10 resulted in a pronounced increase in intracellular mean fluorescence intensity (*P* < 0.01), while DFO significantly restored this trend (*P* < 0.01, Fig. 4N, O). Finally, we observed the fluorescence images of the FerroOrange probe inside the cells and noticed a distinct decrease in fluorescence intensity in the shANXA10 group of cells after DFO treatment (Fig. 4P).

### Knockdown of ANXA10 inhibits autophagy-mediated TFRC degradation

**Fig. 5:**
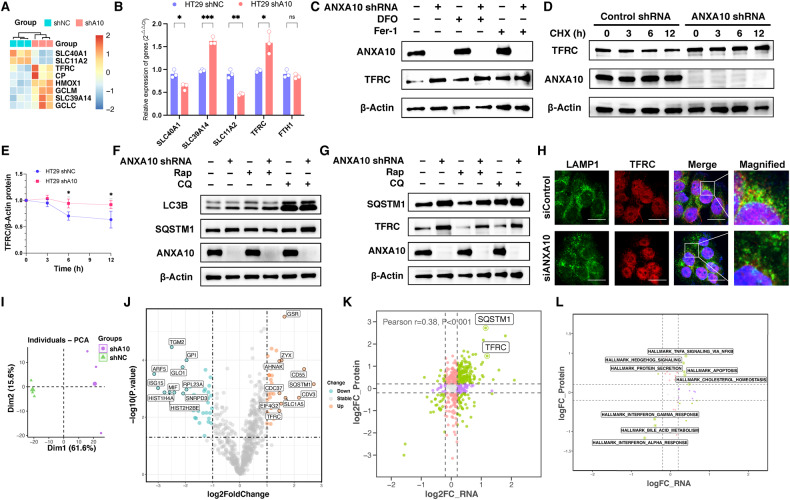
Knockdown of ANXA10-induced ferroptosis through inhibiting autophagy-mediated TFRC degradation. **A** Heatmap showing the expression of genes related to ferroptosis in RNA-seq. The redder the color of the box, the higher the expression of a gene in that sample; conversely, the bluer the color, the lower the expression. **B** qPCR to detect the expression levels of SLC40A1, SLC39A14, SLC11A2, TFRC, and FTH1 mRNA in HT29-shANXA10 and control groups of cells (*n* = 3). **C** Protein expression levels of TFRC in HT29-shANXA10 cells and control were compared by Western blot assay after 48 h incubation in complete medium, complete medium containing DFO (200 μM) or Fer-1 (1 μM). **D**, **E** Western blot assay comparing the protein expression levels of intracellular TFRC in shANXA10 HT29 cells and control after treatment with CHX (50 μg/mL) for 0, 3, 6, and 12 h, respectively (**D**). The TFRC/β-Actin ratio was calculated for both groups after 3, 6, and 12 h of CHX treatment, respectively, using the 0 h group as a reference (**E**). **F** Western blot assay comparing the protein expression levels of LC3B in HT29-shANXA10 cells and control group after 48 h incubation in DMSO (1:1000), Rap (200 nM), or CQ (50 μM). **G** Western blot assay comparing the protein expression levels of SQSTM1 and TFRC in HT29-shANXA10 cells and controls. **H** HT29 cells transfected with control or ANXA10 siRNAs were used for co-localization analysis for LAMP1 and TFRC. Yellow fluorescence in merged images indicates their co-localization. Scale bars = 20 μm. **I** Results of PCA analysis of the proteomics of HT29-shANXA10 and control groups. **J** Volcano plot of DEP. The names of the 20 proteins with the largest |log2FoldChange|values are marked. **K** Correlation analysis of transcriptomic and proteomic results. The horizontal and the vertical coordinates are the results of taking the logarithm of the fold-change in mRNA and protein expression levels, respectively. **L** Correlation analysis of results for statistically significant features identified by GSVA analysis of transcriptomics and proteomics separately for hallmark. The horizontal and the vertical coordinates are the results of logarithmic ploidy change in transcriptomic and proteomic sequencing, respectively. *P < 0.05, **P < 0.01, ***P < 0.001, ns no significance. DEP differentially expressed proteins, DFO deferoxamine mesylate, Fer-1 Ferrostatin-1, Rap rapamycin, CQ chloroquine, CHX cycloheximide.

To dissect the specific mechanisms underlying ferroptosis, we analyzed changes in ferroptosis-related genes in HT29 cells with ANXA10 knockdown. Ligase Catalytic Subunit (GCLC), Glutamate-Cysteine Ligase Modifier Subunit (GCLM), Solute Carrier Family 39 Member 14 (SLC39A14/Zip14), and Ceruloplasmin (CP) were elevated in HT29-shANXA10 cells, while SLC40A1 and SLC11A2/DMT1 were reduced (Fig. 5A). TFRC is a crucial bridge involved in translocating transferrin (TF)-bound iron (III) into cells [27]. Fe^3+^ entering the cell is reduced to Fe^2+^ by STEAP3, a metal reductase in the endosome, and then transported to the cytoplasm by SLC11A2. SLC40A1 is the primary channel for Fe^2+^ export from the cell membrane [28] while SLC39A14 is involved in the uptake of cellular non-transferrin-bound iron [29, 30].

To confirm the changes in iron ion transport-related genes, we examined their mRNA levels in HT29-shANXA10 and ANXA10-knockdown HCT116 cells (Fig. 5B and S3). qPCR results showed that the expression level of TFRC (*P* < 0.05 and *P* < 0.001 in HT29 and HCT116 cells, respectively), which mediates the inward flow of iron ions, was significantly increased in the ANXA10-knockdown groups of cells, whereas the expression levels of SLC40A1 (*P* < 0.05 in both HT29 and HCT116 cells), which translated the inward flow of iron ions, had slightly lower expression levels. Next, we investigated the expression levels of TFRC, an essential protein that is the dominant mode of iron uptake for cells. Western blot assays confirmed higher TFRC protein expression in HT29-shANXA10 cells than in shNC cells. However, the increase in TFRC expression was not restored after 48 h treatment with Fer-1 (1 μM) or DFO (200 μM) (Fig. 5C). The half-life of TFRC protein was significantly longer in HT29-shANXA10 cells than in shNC cells after inhibition of intracellular protein synthesis by cycloheximide (CHX) treatment (*P* < 0.05, Fig. 5D, E). Therefore, we speculate that increased levels of TFRC expression may be responsible for the ferroptosis that occurs in HT29-shANXA10 cells, and the knockdown of ANXA10 inhibits the degradation of TFRC protein in HT29 cells.

**Fig. 6:**
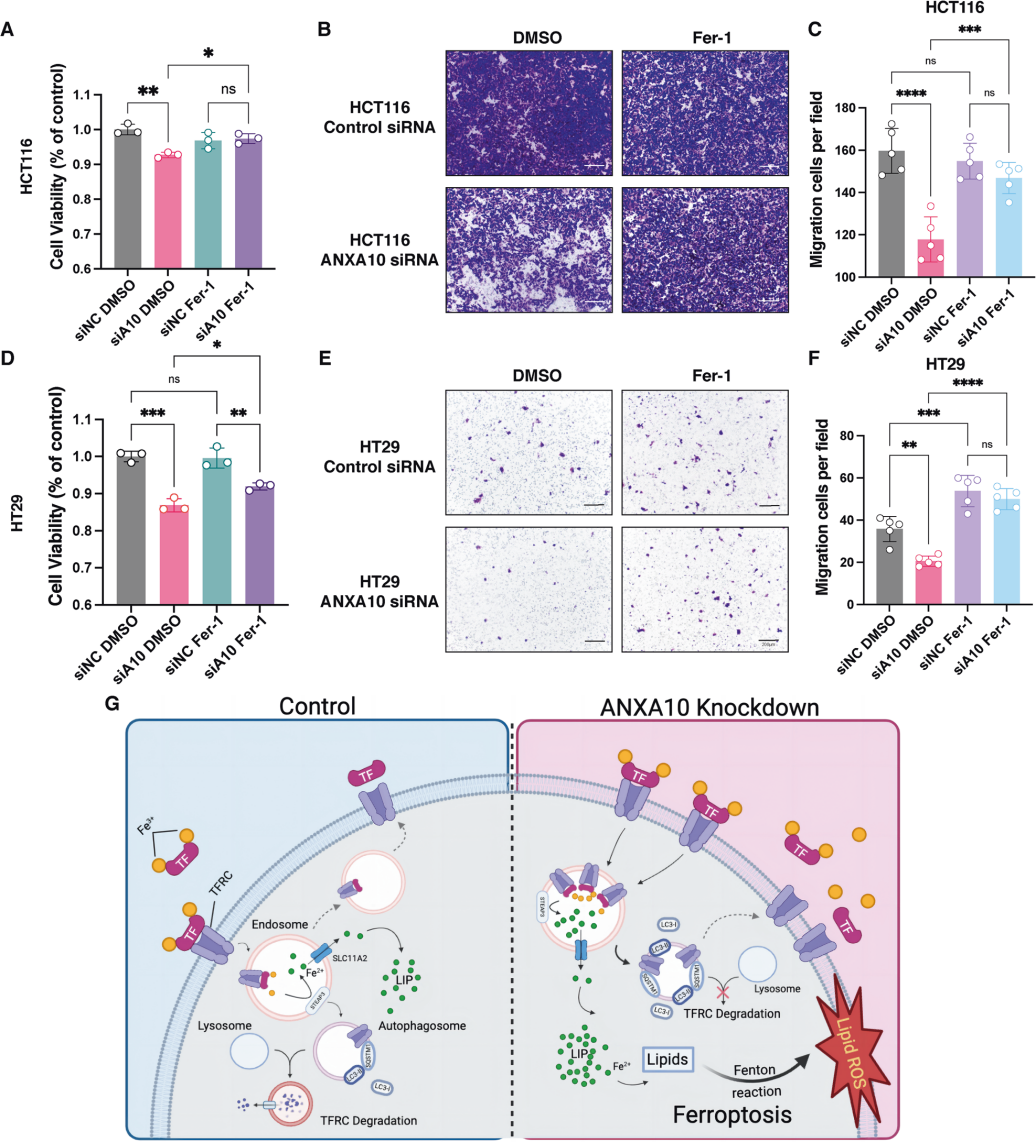
Ferroptosis inhibitor restored viability and migration of colorectal cancer cells with ANXA10 knockdown. **A** CCK-8 assay to detect the cell viability of HCT116 siANXA10 and siNC cells after 48 h incubation in a complete medium containing DMSO (1:1000) and Fer-1 (1 μM), respectively. **B**, **C** Transwell migration assay to detect the effect of Fer-1 (1 μM) on the migration ability of HCT116 siANXA10 cells and siNC cells. **D** CCK-8 assay to determine the cell viability of HT29 siA10 and siNC cells after 48 h incubation in a complete medium containing DMSO (1:1000) and Fer-1 (1 μM) respectively. **E**, **F** Transwell migration assay to detect the effect of Fer-1 (1 μM) on the migration ability of HT29 siANXA10 cells and siNC cells. Five fields of view were randomly photographed in each group at a scale of 200 μm (*n* = 5). **G** The schematic representation of ferroptosis induced by knockdown of ANXA10 through inhibition of autophagy-mediated TFRC degradation. *P < 0.05, **P < 0.01, ***P < 0.001, ****P < 0.0001, ns no significance.

Research has revealed that TFRC degradation can occur through autophagy [31, 32], and based on this, we hypothesized that inhibiting autophagy-mediated degradation through the knockdown of ANXA10 may prevent the degradation of TFRC. To test our conjecture, we examined LC3B and autophagy receptor SQSTM1 protein levels. We found that HT29-shANXA10 cells accumulated significantly higher levels of LC3B compared to control cells, with no change in trend after treatment with either the autophagy inducer rapamycin (Rap) or the autophagy inhibitor chloroquine (CQ) (Fig. 5F). The protein expression level of SQSTM1 was significantly higher in HT29-shANXA10 cells than in the control group (Fig. 5F, G). The immunofluorescence analysis showed that TFRC was co-localized with the lysosomal marker LAMP1 in HT29 cells, but the co-localization of TFRC and LAMP1 decreased after ANXA10 knockdown (Fig. 5H). These results suggest that induction of autophagy partially restored TFRC degradation in HT29-shANXA10 cells, implying that knockdown of ANXA10 prevented TFRC degradation by inhibiting the autophagic process, especially the degradation of TFRC in the lysosome, thereby inducing ferroptosis in CRC cells (Fig. 6G).

To gain a more comprehensive understanding of the ANXA10-regulated protein profile, we performed mass spectrometry analysis on HT29-shANXA10 cells and controls. PCA analysis showed a distinguished difference in protein expression profiles between the two groups (Fig. 5I). Differentially expressed proteins (DEP) were screened according to the criteria of differential expression fold (Fold-Change value) >2-fold or <0.5-fold and *P* < 0.05. 46 proteins with upregulated expression and 40 with down-regulated expression were identified in the HT29-shANXA10 group compared to the control group (Fig. 5J). Correlation analysis of transcriptome and proteome sequencing results revealed that their changes were positively correlated after ANXA10 knockdown (Pearson *r* = 0.38, *P* < 0.001, Fig. 5J). Interestingly, SQSTM1 and TFRC were among the 33 genes with significant changes in both histological sequencing results (*P* < 0.05) (Fig. 5K), confirming that knockdown of ANXA10 led to the accumulation of SQSTM1 and suppression of TFRC degradation in CRC cells. Moreover, correlation analysis of transcriptomic and proteomic significantly enriched Hallmark pathways revealed that characteristic pathways such as tumor necrosis factor α (TNF-α)/NFκB signaling, apoptosis, Hedgehog signaling, protein secretion, and cholesterol homeostasis were significantly enriched in the shANXA10 group, while pathways such as interferon γ responsiveness, interferon α responsiveness, and bile acid metabolism were downmodulated (Fig. 5L).

### Cell viability and migration were rescued by Ferrostatin-1 in ANXA10 knockdown colorectal cancer cells

Owing to the above observations, we hypothesized that the knockdown of ANXA10 induces ferroptosis in CRC cells by inhibiting autophagy-mediated TFRC degradation, thereby suppressing cell survival and migration ability. Our results demonstrated that treatment with Fer-1 restored the decreased cell viability of HCT116 and HT29 cells caused by ANXA10 knockdown (Fig. 6A, D), while having no significant effect on the survival of control cells. Fer-1 greatly improved the migration ability of ANXA10 knockdown HCT116 (Fig. 6B, C) and HT29 cells (Fig. 6E, F). These findings collectively showed that the ferroptosis inhibitor recovered the survival and migration capacity of knockdown ANXA10 CRC cells.

## Discussion

ANXA10, a calcium-dependent phospholipid-binding protein, has been linked to poor survival in CRC [33] and an enhanced risk of developing heterochronic serrated lesions in individuals with serrated lesions [12]. This study is the first to investigate the expression and mechanisms of ANXA10 in the serrated carcinoma pathway of CRC. Consistent with previous studies, ANXA10 is specifically highly expressed in SSL and *BRAF*mut CRC and is associated with poor prognosis. single-cell sequencing and immunohistochemical assays confirmed that ANXA10 was predominantly expressed in epithelial cells with serrated lesions.

Research on ferroptosis is expanding and showing potential for the development of new therapeutic approaches for CRC. For example, salazosulfapyridine can induce ferroptosis by inhibiting the cell membrane cystine glutamate transporter receptor (System Xc-) and depleting GSH, which promotes sensitivity to cisplatin in CRC [34]. Dimethyl fumarate induces ferroptosis in CRC cells by depleting GSH, increasing ROS, and activating MAPK [35]. Targeting ferroptosis can enhance the therapeutic effect of CRC, improve sensitivity to radiotherapy, and reduce cellular resistance [36]. Our study found that knockdown of ANXA10 induced ferroptosis in CRC cells, suggesting that ANXA10 is an effective target for inducting ferroptosis in the treatment of ANXA10 highly expressed CRC.

Iron deficiency in CRC has been linked to a less favorable prognosis and poor treatment response [37, 38]. Therefore, increasing the concentration of Fe^2+^ in CRC cells is expected to improve the prognosis of CRC patients. Our study revealed that knockdown of ANXA10 inhibited the degradation of TFRC, resulting in a significant increase in intracellular Fe^2+^ concentration and inducing ferroptosis in CRC cells. Thus, it is possible to significantly inhibit cell growth by up-regulating the concentration of iron ions in ANXA10 highly expressed CRC cells.

In our study, we observed a distinct increase in autophagic flux and accumulation of SQSTM1 following knockdown of ANXA10, which ultimately hindered the autophagic process in CRC cells, suggesting that ANXA10 plays a role in the autophagy process. This biological function is similar to other proteins of the membrane-linked protein A family [11], such as ANXA2, which promotes the formation of autophagosomes and mediates the fusion of autophagosomes and lysosomes [39, 40]. The role played by ANXA10 in the autophagic process warrants further investigation.

While our findings showed that the knockdown of ANXA10 induced ferroptosis and hindered the malignant proliferation and migration of CRC cells, it remains unclear whether the ferroptosis triggered by ANXA10 downregulation can enhance the sensitivity of existing chemotherapy, targeted therapy, or immunotherapy regimens. Therefore, it is necessary to conduct cellular and animal-level experiments to demonstrate the efficacy of ANXA10 knockdown in combination with other therapeutic regimens. In addition, further studies should focus on the mechanisms by which ANXA10 affects TFRC degradation in lysosomes and understand how ANXA10 regulates the autophagy cycle.

This study provides strong evidence that ANXA10 is significantly upregulated in patients with SSL and *BRAF* mutant CRC, and is strongly associated with poor prognosis. Our results indicate that knockdown of ANAX10 can inhibit the malignant capacity of colorectal cancer cells by inhibiting autophagy-mediated degradation of TFRC, leading to cellular ferroptosis. The ANXA10–TFRC–ferroptosis axis sheds light on a novel therapy for CRC originating from the serrated pathway. Further exploration of this axis could potentially lead to the development of more effective treatment options for this subtype of CRC, ultimately improving patient outcomes.

### Supplementary information

Supplementary information

Figure S1

Figure S2

Figure S3

Table S1 Primer sequences for siRNA and shRNA.

Table S2 Primer sequences for qPCR.

Table S3 Proteome sequencing data.

reproducibility checklist

Original Data File

## Data Availability

The datasets generated during the current study are available in the NIH National Center for Biotechnology Information Sequence Read Archive (SRA) repository, http://www.ncbi.nlm.nih.gov/bioproject/950129 and Supplementary Table S3.
