# Publisher Correction: Knockdown of ANXA10 induces ferroptosis by inhibiting autophagy-mediated TFRC degradation in colorectal cancer

**DOI:** 10.1038/s41419-023-06151-x

**Published:** 2023-10-16

**Authors:** Xinyuan Wang, Yujie Zhou, Lijun Ning, Jinnan Chen, Huimin Chen, Xiaobo Li

**Affiliations:** Division of Gastroenterology and Hepatology, Shanghai Institute of Digestive Disease, NHC Key Laboratory of Digestive Diseases, State Key Laboratory for Oncogenes and Related Genes, Renji Hospital, School of Medicine, Shanghai Jiao Tong University, Shanghai, China

**Keywords:** Colon cancer, Diagnostic markers, Tumour biomarkers

Correction to: *Cell Death and Disease* 10.1038/s41419-023-06114-2, published online 04 September 2023

Due to a technical problem the uncorrected proof has been published online. The online PDF version of this article has been updated now.

