# Supplementary material for: Knockdown of ANXA10 induces ferroptosis by inhibiting autophagy-mediated TFRC degradation in colorectal cancer
Source: Cell Death Dis. 2023 Sep 4;14(9):588. doi: 10.1038/s41419-023-06114-2 (PMC10477278; doi:10.1038/s41419-023-06114-2)
Supplement: Supplementary file 1 — Supplementary information [file 41419_2023_6114_MOESM1_ESM.docx]

Figure S1 Validation of ANXA10 knockdown and overexpression efficiency.

(A) RT-PCR and western blot to detect the efficiency of different siRNAs on ANXA10 knockdown in HT29 cells. The left panel shows the mRNA level and the right panel shows the protein level results. (B) RT-PCR and western blot to detect the effect of siRNA-2 on ANXA10 knockdown in HCT116 cells. The upper panel shows the mRNA level, and the lower panel shows the protein level results. (C) RT-PCR and western blot to detect the expression level of ANXA10 in shRNA-constructed HT29-shANXA10 cells. The upper panel shows the mRNA level and the lower panel shows the protein level results. (D-E) RT-PCR and western blot to detect the effect of transfection of overexpressed ANXA10 plasmid in SW480 (D) and RKO (E) cells. The upper panel shows the mRNA level, and the lower panel shows the protein level results. **P* < 0.05,***P* < 0.01,****P* < 0.001,*****P* < 0.0001.

Figure S2 Overexpression of ANXA10 inhibited apoptosis in CRC cells.

(A-B) Flow diagram of the change in the percentage of apoptotic cells after knockdown of ANXA10 in HCT116 cells using flow cytometry (A) and the statistical results of the percentage of early apoptosis + late apoptosis (Q2+Q3) (B). (C-F) Flow diagram of the change in the percentage of apoptotic cells after overexpression of ANXA10 in SW480 and RKO cells using flow cytometry (C and E) and the statistical results of the percentage of early apoptosis + late apoptosis (Q2+Q3) (D and F). (G-H) Flow diagram of the change in the percentage of apoptotic cells after overexpression of ANXA10 and treatment with 5ug/ml CHX for 24 h in RKO cells using flow cytometry (G) and the statistical results of the percentage of early apoptosis + late apoptosis (Q2+Q3) (H). **P* < 0.05, ***P* < 0.01. CHX: cycloheximide

Figure S3 The expression levels of iron ion transport-related genes in ANXA10-knockdown HCT116 cells.

(A) qPCR to detect the expression levels of SLC40A1, SLC39A14, SLC11A2, TFRC and FTH1 mRNA in HCT116 siANXA10 and control groups of cells (n=3). **P* < 0.05, ****P* < 0.001, *****P* < 0.0001, ns: no significance.

Table S1 Primer sequences for siRNA and shRNA.

Table S2 Primer sequences for qPCR.

Table S3 Proteome sequencing data.
