# Supplementary figures and images for: Knockdown of ANXA10 induces ferroptosis by inhibiting autophagy-mediated TFRC degradation in colorectal cancer
Source: Cell Death Dis. 2023 Sep 4;14(9):588. doi: 10.1038/s41419-023-06114-2 (PMC10477278; doi:10.1038/s41419-023-06114-2)

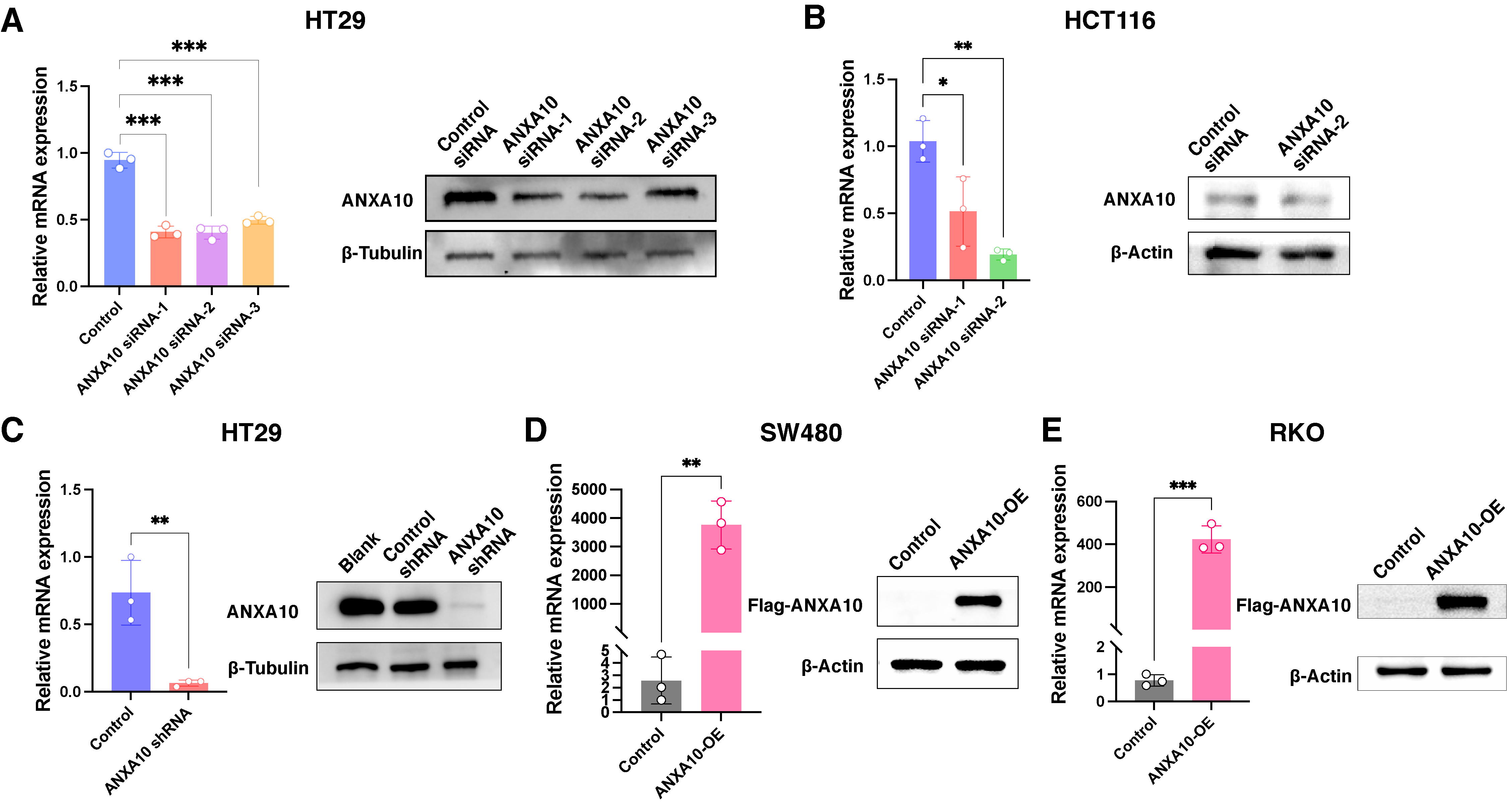

Supplement: Supplementary file 2 — Figure S1 [file 41419_2023_6114_MOESM2_ESM.tif]

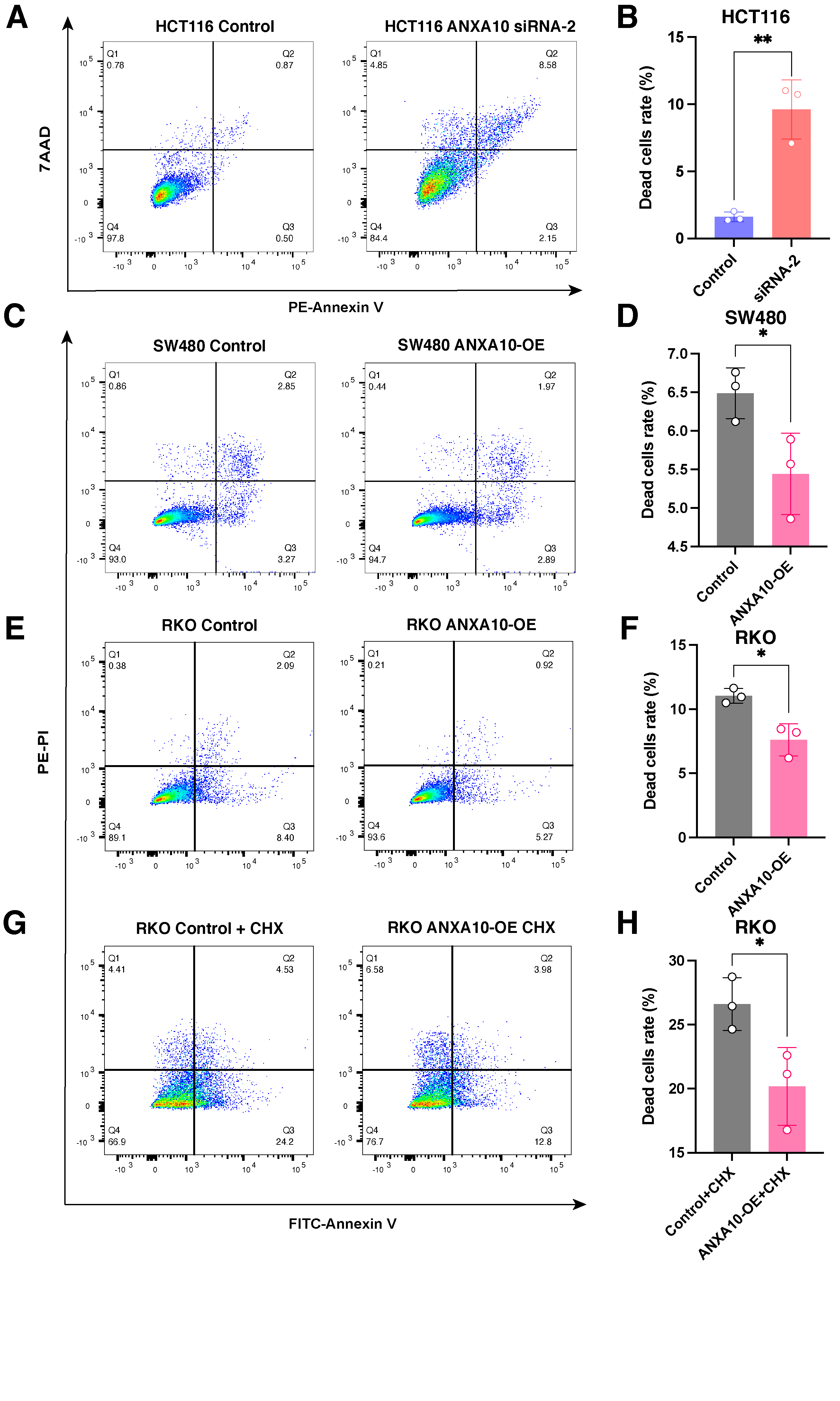

Supplement: Supplementary file 3 — Figure S2 [file 41419_2023_6114_MOESM3_ESM.tif]

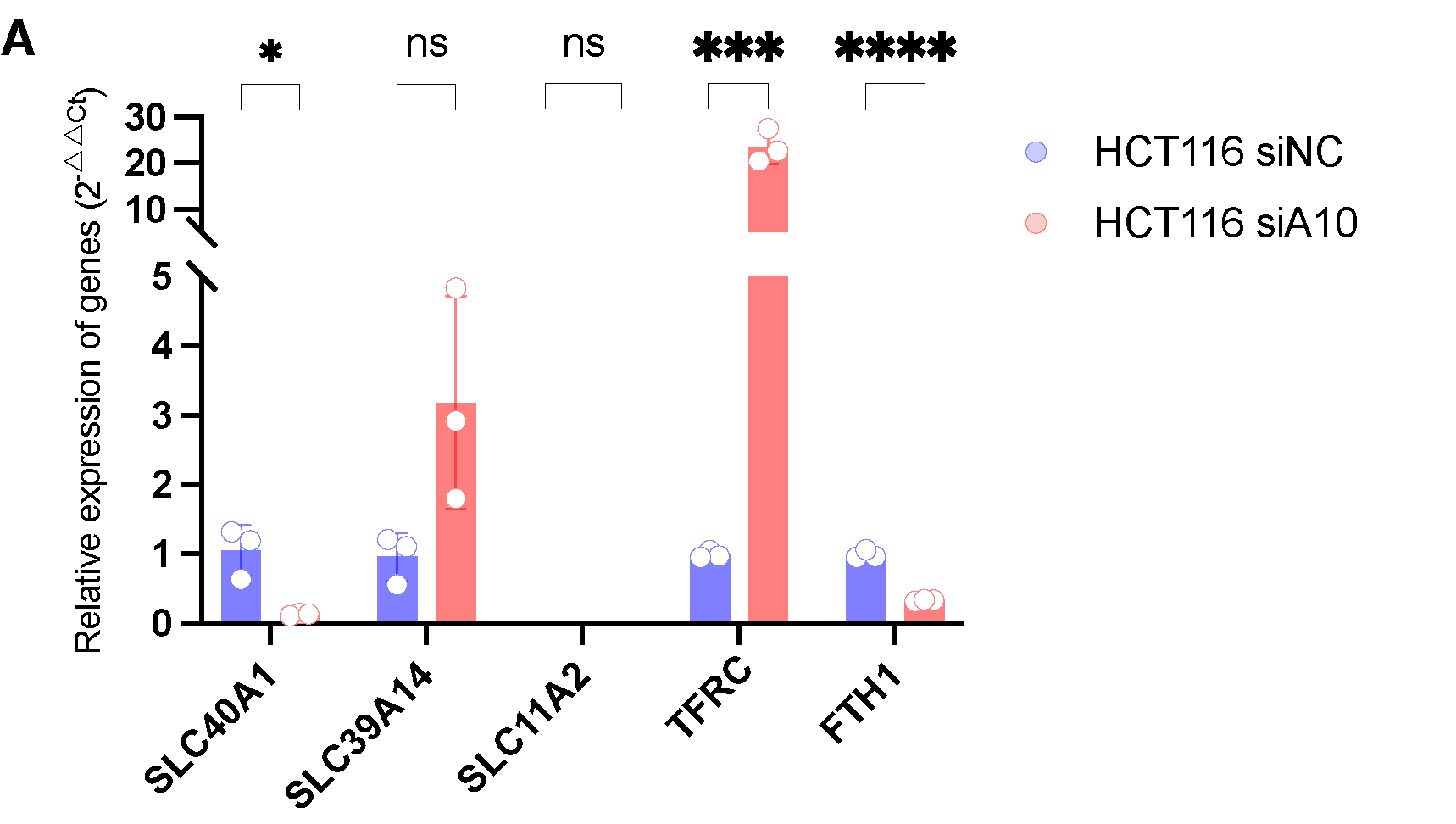

Supplement: Supplementary file 4 — Figure S3 [file 41419_2023_6114_MOESM4_ESM.tif]

Figure 5

C

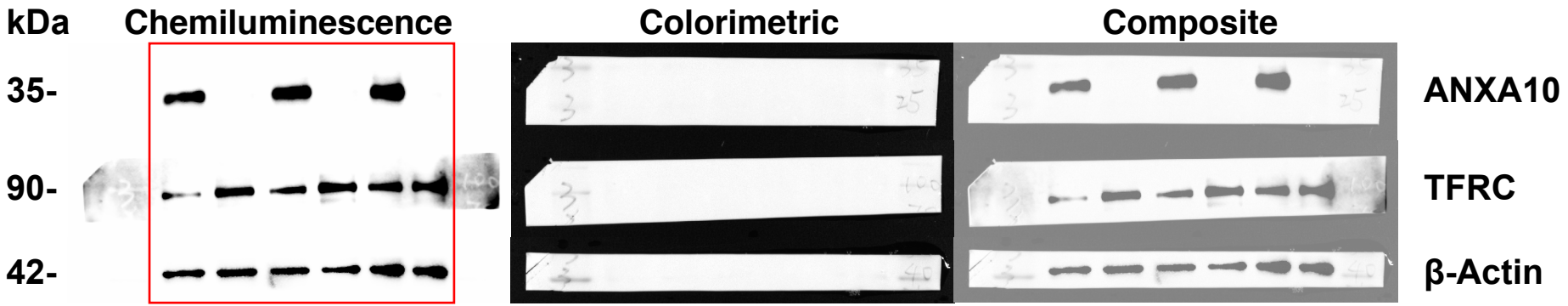

D

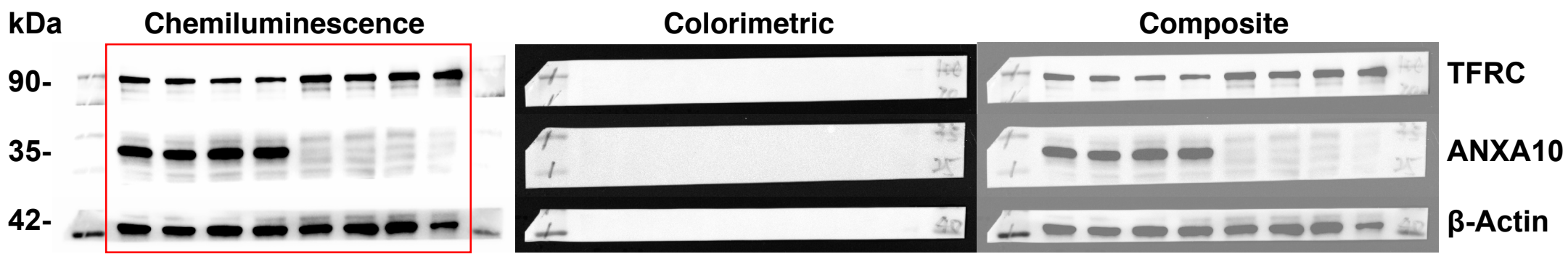

F

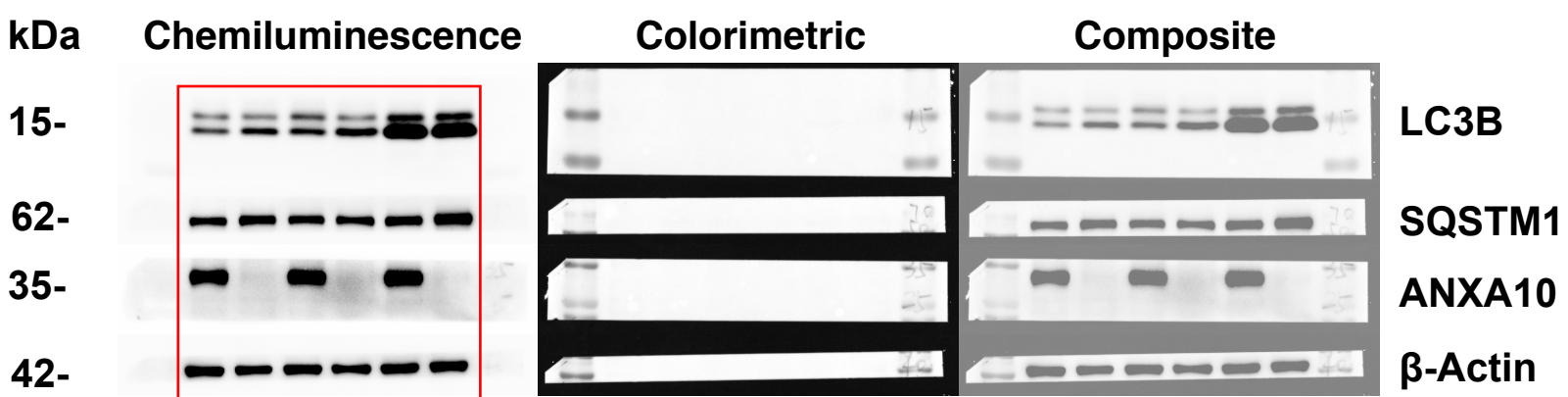

G

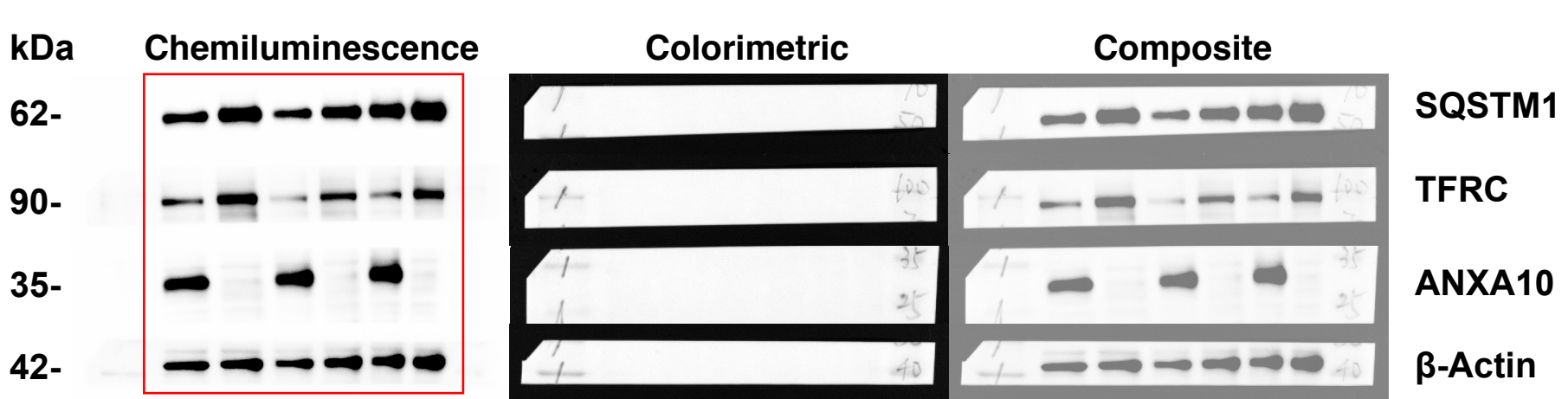

**Figure S1**

**A**

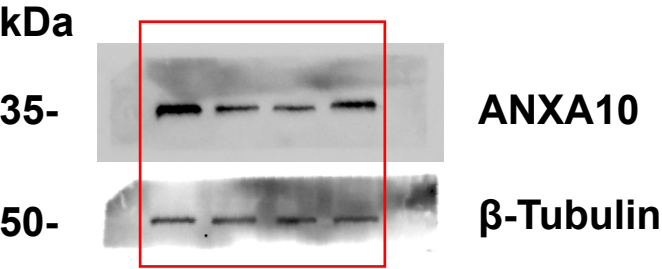

**B**

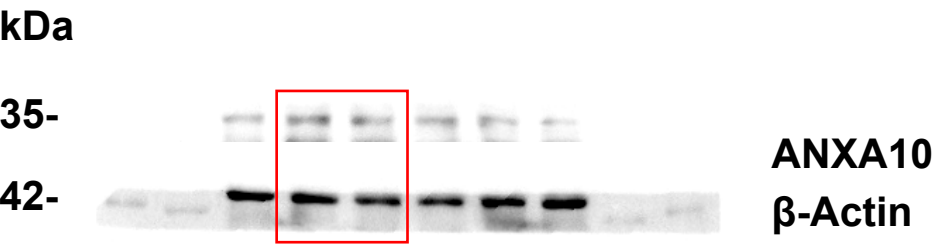

**C**

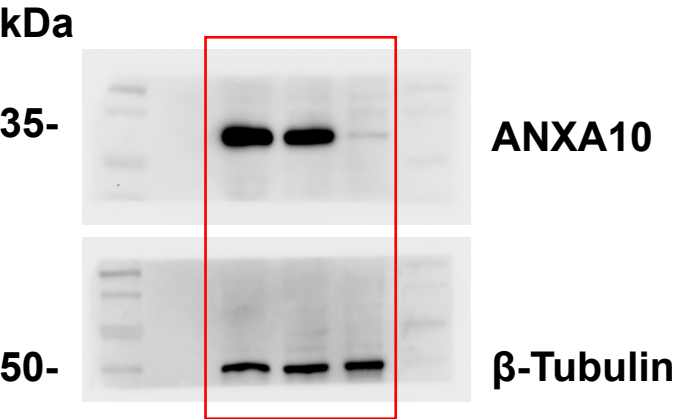

**D**

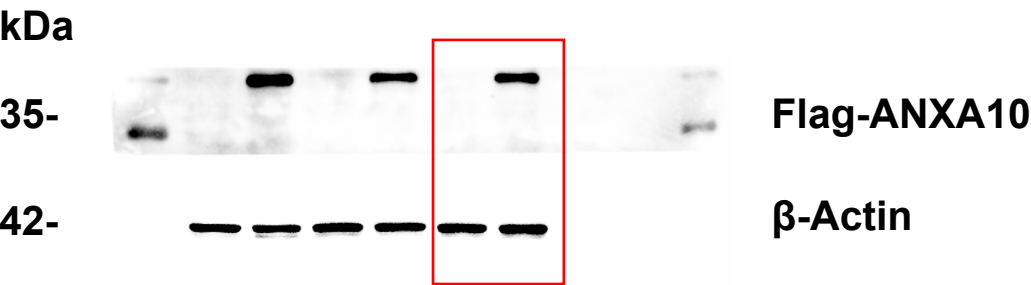

**E**

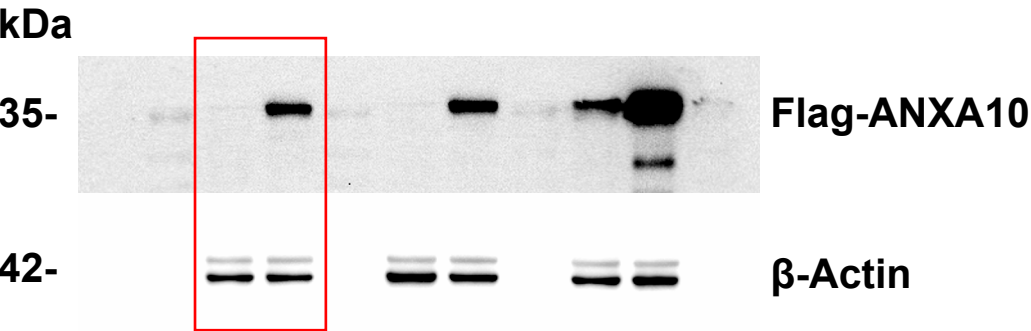

Supplement: Supplementary file 9 — Original Data File [file 41419_2023_6114_MOESM9_ESM.pdf]
