# Supplementary material for: Knockdown of ANXA10 induces ferroptosis by inhibiting autophagy-mediated TFRC degradation in colorectal cancer
Source: Cell Death Dis. 2023 Sep 4;14(9):588. doi: 10.1038/s41419-023-06114-2 (PMC10477278; doi:10.1038/s41419-023-06114-2)
Supplement: Supplementary file 5 — Table S1 Primer sequences for siRNA and shRNA. [file 41419_2023_6114_MOESM5_ESM.docx]

Table S1 Primer sequences for siRNA and shRNA.

| Gene | Sense（5’→3’） | Antisense（5’→3’） |
| --- | --- | --- |
| Negative Control | UUCUCCGAACGUGUCACGUTT | ACGUGACACGUUCGGAGAATT |
| ANXA10 siRNA-1 | GGAGCUGCUGGUUGCAAUUTT | AAUUGCAACCAGCAGCUCCTT |
| ANXA10 siRNA-2 | CAAGGAUUUGACUGUGACAAA | UUUGUCACAGUCAAAUCCUUG |
| ANXA10 shRNA | CCGGCAAGGATTTGACTGTGACAAACTCGAGTTTGTCACAGTCAAATCCTTGTTTTTG | AATTCAAAAACAAGGATTTGACTGTGACAAACTCGAGTTTGTCACAGTCAAATCCTTG |
