# Supplementary material for: Knockdown of ANXA10 induces ferroptosis by inhibiting autophagy-mediated TFRC degradation in colorectal cancer
Source: Cell Death Dis. 2023 Sep 4;14(9):588. doi: 10.1038/s41419-023-06114-2 (PMC10477278; doi:10.1038/s41419-023-06114-2)
Supplement: Supplementary file 6 — Table S2 Primer sequences for qPCR. [file 41419_2023_6114_MOESM6_ESM.docx]

Table S2 Primer sequences for qPCR.

| Gene | Sense（5’→3’） | Antisense（5’→3’） |
| --- | --- | --- |
| ANXA10 | CAGAGACCTCAGGACACTTCAG | TCTGCTGACAGGCTTCCCATAG |
| β-ACTIN | CACCATTGGCAATGAGCGGTTC | AGGTCTTTGCGGATGTCCACGT |
| SLC40A1 | CTACTTGGGGAGATCGGATGT | CTGGGCCACTTTAAGTCTAGC |
| SLC39A14 | GGACGAGAAGGTCATTGTGG | GTGATCATCCAGGCCAGAGT |
| SLC11A2 | CCATATGAAATATAAAATGAAGAGACACCTA | CCCCTCTTAACTTCCACTGAGAAA |
| TFRC | CGTGATCAACATTTTGTTAAGATTCA | CCACATAACCCCCAGGATTCT |
| FTH1 | GGAACATGCTGAGAAACTGATGAA | CATCACAGTCTGGTTTCTTGATATCC |
